# Collision-constrained deformable image registration framework for discontinuity management

**DOI:** 10.1371/journal.pone.0290243

**Published:** 2023-08-18

**Authors:** Thomas Alscher, Kenny Erleben, Sune Darkner

**Affiliations:** Department of Computer Science, University of Copenhagen, Copenhagen, Region Hovedstaden, Denmark; VIT University, INDIA

## Abstract

Topological changes like sliding motion, sources and sinks are a significant challenge in image registration. This work proposes the use of the alternating direction method of multipliers as a general framework for constraining the registration of separate objects with individual deformation fields from overlapping in image registration. This constraint is enforced by introducing a collision detection algorithm from the field of computer graphics which results in a robust divide and conquer optimization strategy using Free-Form Deformations. A series of experiments demonstrate that the proposed framework performs superior with regards to the combination of intersection prevention and image registration including synthetic examples containing complex displacement patterns. The results show compliance with the non-intersection constraints while simultaneously preventing a decrease in registration accuracy. Furthermore, the application of the proposed algorithm to the DIR-Lab data set demonstrates that the framework generalizes to real data by validating it on a lung registration problem.

## Introduction

Inadequate management of discontinuities in the displacements field of image registration causes problems for widely-used algorithms utilizing smoothness regularizations [1]. One such instance is sliding motions along domain boundaries as observed in longitudinal registration of lungs. Algorithms producing a single smooth deformation field cannot account for discontinuities. A separation of the registration domain into its sliding segments is, thanks to increasingly well-performing deep learning methods for accurate individual organ segmentation [2–4], available but subsequent, independent registration of the resulting segments suffers from the possible overlap of the computed deformation fields. In order to acquire separate and congruent deformation fields without intersection, an additional constraining mechanism is required. To this end, the alternating direction method of multipliers (ADMM) is presented to enforce constraints preventing overlap of the individual components.

Extending the early development of rigid [5] registration algorithms, the implementation of diffusion inspired processes [6], called DEMONs, as well as non-rigid deformations introduced by Free-Form Deformations (FFD)[7] account for more complex image registrations. Analogies from fluid mechanics allowed to consider large deformations [8, 9]. Topology preservation in image registration is enforced by the use of diffeomorphisms. The combination with the large deformation algorithms gave rise to another approach, called Large Deformation Diffeomorphic Metric Mapping (LDDMM)[10, 11]. One type of discontinuities, particularly present in lung and abdominal registrations, are sliding motions [12]. The sliding of the lung along the pleura and the expansion of the rib cage in the opposite direction leads to severe singularities in the deformation field and consequently invalidate the usual premise of smooth displacement fields [13].

Existing approaches aiming to deal with sliding motions can be categorized into methods computing a single deformation field for the whole image domain and those that split the domain into individual parts for each independently moving subdomain and subsequently computing as many deformation fields. Solutions computing one deformation field require additional constraints controlling smoothing across domain boundaries. One such approach of enriching the b-spline basis functions with additional information around discontinuities, i.e. in the lung case domain boundaries, is proposed in [14] and prevents information exchange over domain boundaries. Due to its built, the approach doesn’t require further regularization or a specific interface smoothness. Being a completely intensity based method without explicitly penalizing overlaps, the image gradient may push voxel over the domain boundary regardless of the underlying enrichment. Further approaches utilizing the need for regularization differentiation between sub-domains can be divided into two groups [13]: direction-dependent methods and locally adaptive methods. Direction-dependent approaches decompose the deformation field into tangential and normal components. Smoothing the image globally only in the direction of the surface normals and tangentially only inside segmented domains offers a closed mathematical solution, with the draw back of stationary normal directions [15]. Using a piece wise diffeomorphic solution with a stationary velocity field implementation that smooths the velocity field domain independently in tangential direction while matching the velocity direction and magnitude on both sides of the domain interface is introduced in [16]. However, using different resolutions in different domains requires interpolation at the domain boundary effectively smoothing intensities. Locally adaptive approaches implement regularization whose weight in the loss function differs according to the location in the image domain. The first locally adaptive version investigated in this section offers to balance diffusion-based *L*_2_ norm regularization for smooth domain interiors and *L*_1_ norm total variation at domain boundaries, showing good results even with minor domain segmentation errors. Low magnitude updates, e.g. small scale sliding motion, can still be falsely smoothed [17]. [18] uses a classical bilateral filtering kernel to identify interface boundaries with the application of a computationally costly kernel. An alternative to bilateral filtering is isotropic total variation [1]. Both methods, bilateral filtering and total variation are challenged, however, by interfaces between similarly textured and low contrast domains. Registration algorithms splitting the image domain into independently moving sub-domains suffer more than the methods above from possible overlaps or gaps in between distinct deformation fields. [19] minimizes the overlaps by assigning unique, penalizing intensities for voxel lying outside the respective sub-domain during registration, allowing for the use of different registration parameters for each sub-domain. The definition of the right intensities can be tedious and time consuming. [20–22] drop intensity based penalization in favour if a distinct term added to the loss function penalizing overlaps and gaps. These terms can consist of the product of deformed signed distance fields [20], requiring a computationally expensive creation of motion mask and being restricted to two sub-domains, or a local distance metric to the opposing interface via the sum of sample surface points [22]. The second implementation offers a solution to sliding along curved surfaces and surfaces that are separated by a third domain, but relies on the resolution of the sampled surface points. Another approach penalizing a set of sampled surface points is the split Gauss-Newton approach [21, 23]. Here any deviation of the point set from the deformed interface is weighted relative to the distance to the interface. If not parametrized properly, the penal term can overrule the registration forces, keeping the deformation field in its initial state. Using not *n* deformation fields for as many sub-domains, but *n* + 1, [24] utilizes the additional field to calculate the global normal displacements only. The normal direction is derived from local bases. The remaining deformation fields produce the sub-domain specific tangential displacements, ensuring matching normal displacements along the interface. This approach assumes smooth, small-deforming interfaces, since the bases are created in the moving image only. All aforementioned methods except [1, 18] require correct segmentations.

This work presents a registration framework, the Collision Constrained Deformable Image Registration (CC-DIR), to account for sliding motions by combining a collision constraining regularization with the registration term and solving the resulting minimization problem using the ADMM.

## Materials and methods

### Image registration

Formally, in image registration a transformation Φ: Ω → Ω is sought that aims to align the source or moving image *I* ∈ *R*^*n*^ via the transformation Φ ∈ Ω to a target or fixed image *J* ∈ *R*^*n*^. The registration problem can be written as a minimization problem of the energy term *F*: Ω → *R*

$$\begin{array}{c} \underset{\Phi }{\text{min}\;}F\left(\Phi ,I,J\right)=\underset{\Phi }{\text{min}\;}\;M\left(J,I\circ \Phi \right)+S\left(\Phi \right).\; \end{array}$$ (1)

The image similarity *M*: Ω → *R* measures the alignment between two images. Difference methods like Sum of Squared Differences (SSD) and Sum of Absolute Differences (SAD) are widely used as similarity measures for monomodal registrations that map the same anatomical structures, performing similarly well [25]. SSD methods penalize outliers in intensity heavier [26] and are in consequence a valid approach to properly register the relative high differences between lung tissue and the surrounding air. Image registration as an ill-posed problem has no single solution [27] and often utilizes regularization *S*: Ω → *R*. Regularization may be applied for smoothing [28] or constraining the deformation to reasonable [29] solutions. Cubic B-splines utilized in FFD act as a smoothing alternative themselves [28] and free the algorithm of additional regularization implementations.

### Parametrization

Parametrizations offer a reduction in dimensionality, improved robustness [1], and decreased risk of overfitting. One example are Free-Form deformations. By deforming an overlying grid of control points, the deformation of the underlying image is calculated by interpolating the pixel- or voxelwise displacements. Widely used approximation functions for individual voxel displacements are cubic B-Splines, which have a very local influence and only affect points in their neighbourhood [7]. For an exemplary image in *R*^3^, $\zeta \in R^{n_{1}\times n_{2}\times n_{3}}$ is the mesh control points spaced equidistantly at *δ* = (*δ*_1_, *δ*_2_, *δ*_3_). The displacement of *p* ∈ *R*^3^

$$\begin{array}{c} \Phi \left(p\right)=\sum_{l=0}^{3}\sum_{m=0}^{3}\sum_{q=0}^{3}B_{l}\left(u\right)B_{m}\left(v\right)B_{q}\left(w\right)\zeta_{i+l,j+m,k+q} \end{array}$$ (2)

with the basis functions [30, 31]

$$B_{0}(u)=\frac{1}{6}{\left(1-u\right)}^{3},$$ (3a)

$$B_{1}(u)=\frac{1}{6}(3u^{3}-6u^{2}+4),$$ (3b)

$$B_{2}(u)=\frac{1}{6}(-3u^{3}+3u^{2}+3u+1),$$ (3c)

$$B_{3}(u)=\frac{1}{6}u^{3}.$$ (3d)

Location of the respective control points follows *i* = ⎥*p*_1_/*δ*_1_⎦ − *ξ*, *j* = ⎥*p*_2_/*δ*_2_⎦−*ξ*, *k* = ⎥*p*_3_/*δ*_3_⎦ − *ξ*, with *ξ* shifting the support along the axis. The localized coordinates derive from *u* = (*p*_1_−*δ*_1_*⎥*p*_1_/*δ*_1_⎦)/*δ*_1_, *v* = (*p*_2_ − *δ*_2_*⎥*p*_2_/*δ*_2_⎦)/*δ*_2_, and *w* = (*p*_3_ − *δ*_3_*⎥*p*_3_/*δ*_3_⎦)/*δ*_3_. By decreasing the grid resolution, cubic b-spline represent global deformations as well. Consequently, a multiresolution approach with sequentially increasing resolutions will capture both local and global deformations.

### Collision detection

Collision detection algorithms can be classified according to utilized object representation [32], such as purely explicit, implicit or hybrid approaches. Explicit representations may range from simple point clouds to polygon meshes, one of the most common tools utilized [33]. Implicit representations, such as signed distance fields $SDF:R^{n}\to R$, describe geometries as a mapping function. A hybrid approach of a point cloud, representing the deformable object, with a rigid object, depicted as a SDF, has been proven to perform fast and accurately [34]. On top, this algorithm leverages existing data structures such as segmentation masks and can be directly pre-computed from segmented images by fast marching methods, allowing for fast intersection tests [33]. This sidesteps the need for object meshing, which proves hard to implement for anatomical structures [35]. Let $p_{i}\in R^{n}$ be any point in the image and $\omega \subset R^{n}$ by a closed and bounded [36] subset representing a collision object with *δω* as its boundary. Detecting a collision of *p*_*i*_ with *ω* requires evaluating the signed distance

$$\begin{array}{c} SDF_{\omega }\left(p_{i}\right)=\{\begin{array}{cc} -d(p_{i}) & p_{i}\in \omega \\ \;\;\;0 & p_{i}\in \delta \omega \\ \;\;\;d(p_{i}) & p_{i}\notin \omega \end{array} \end{array}$$ (4)

with $d:R^{n}\to R$

$$\begin{array}{c} d\left(p_{i}\right)=\underset{y\in \delta \omega }{\text{inf}}{∥p_{i}-y∥}_{{}_{2}} \end{array}$$ (5)

equating to the shortest distance to its domain boundary. The intersections are formulated as a quadratic penalty function $g:R^{n}\to R$ [37].

$$\begin{array}{c} g\left(p_{i}\right)=\{\begin{array}{cc} 0 & SDF_{\omega }\left(p_{i}\right)\geq 0\\ \frac{\mu }{2}SDF_{\omega }{\left(p_{i}\right)}^{2}\;\;\;0 & SDF_{\omega }\left(p_{i}\right)<0 \end{array} \end{array}$$ (6)

This function weights possible collisions by the depth of intersection of colliding particles with respect to the object surface with an adjustable free parameter *μ*. Neglecting all influences of non-intersecting points by simple piece-wise definition *g* acts both as the collision detection and correction, with a value only deviating from 0 once *p*_*i*_ collides with *ω*.

Reformulating this collision constraint for the registration problem to account for sliding motion between the image domains *ω*_1_ ⊂ *I* and its counterpart *ω*_2_ ⊂ *J*, let *X*_*i*_ ∈ *ω*_1_ be the set of all material points in the undeformed configuration. The energy formulation in the deformed configuration *x*_*i*_ = *X*_*i*_ ∘ Φ reads as

$$\begin{array}{c} C\left(\Phi ,I,J\right)=\sum_{i}g\left(x_{i}\right) \end{array}$$ (7)

Here, the $SDF_{\omega_{2}}$ is calculated in spatial space. Adding the collision constraint energy formulation to the initial registration problem changes the term to

$$\begin{array}{c} \underset{\Phi }{\text{min}\;}F\left(\Phi ,I,J\right)+C\left(\Phi ,I,J\right). \end{array}$$ (8)

### Alternating direction method of multipliers

With the constrained registration problem now consisting of *F*(Φ, *I*, *J*) and *C*(Φ, *I*, *J*), we can take advantage of this formulation by splitting the variable **Φ** representing the transformation into two distinct deformations **Φ_*R*_** and **Φ_*C*_** for the registration respectively collison problem and adding a coupling constraint.


$$\begin{array}{c} \begin{array}{cc} \underset{\Phi_{R},\Phi_{C}}{\text{min}\;}F\left(\Phi_{R},I,J\right) & +C(\Phi_{C},I,J)\\ \;\text{subject}\;\text{to}\;\Phi_{R} & -\Phi_{C}=0. \end{array} \end{array}$$ (9)

This formulation clearly illustrates the advantage of applying the ADMM. Utilizing a combination of dual ascent and decomposition, the ADMM splits the objective function into sub-problems, favouring problems where the local optimization of theses sub-problems can be carried out efficiently [38]. The registration problems in the form of Eq 9 can be solved by iterating through the sequential updates of Φ_*R*_, Φ_*C*_, and a so-called dual variable *u* by

$${\vec{\Phi }}_{R}^{k+1}\leftarrow \underset{\Phi_{R}}{\text{arg}\;\text{min}}\;F(\Phi_{R},I,J)+\frac{\rho }{2}{\left\|\Phi_{R}-\Phi_{C}^{k}+u^{k}\right\|}_{2}^{2},$$ (10a)

$${\vec{\Phi }}_{C}^{k+1}\leftarrow \underset{\Phi_{C}}{\text{arg}\;\text{min}}\;C(\Phi_{C},I,J)+\frac{\rho }{2}{\left\|\Phi_{R}^{k+1}-\Phi_{C}+u^{k}\right\|}_{2}^{2},$$ (10b)

$$u^{k+1}\leftarrow u^{k}+\Phi_{R}^{k+1}-\Phi_{C}^{k+1},$$ (10c)

until either convergence or a fixed number of iterations is achieved. This scaled version of the ADMM utilizes *u* as the accumulating sum of residuals for the coupling constraint [39] whereas the free parameter *ρ* allows to control its influence on the sequential updates Convergence towards an optimum is sufficiently fast for moderate accuracy, but may be slow to produce highly accurate results.

## Experiments & results

2D synthetic examples under controlled conditions, as proof of concept, as well as an application to 3D medical data to showcase its feasibility, are conducted.

### Implementation

For each domain *ω*_*r*_ representing an independently moving object in *I* ∈ *R*^*n*^, in this paper in both synthetic and medical experiments *r* ∈ {1, 2}, a CC-DIR is executed. The object is represented as two point clouds of *i* respectively *j* evaluation points for **p**_*col*_, **p**_*reg*_ in the collision respectively registration problem. Cubic B-spline parametrization (Eq 2) is used as the deformation model, while for interpolating voxel intensities at non-integer positions, linear interpolation is utilized. For updating $\Phi_{C}^{N+1},\Phi_{R}^{N+1}$ during the iterations, subsolvers are called to minimize the respective function and assign the minimizing variables Φ_*C*_, **Φ**_*R*_. The subsolvers are gradient descent implementations with a backtracking according to [40], running for 2 iterations. *ρ* is kept at 0.5 across all experiments. In our implementation the stopping criterion for CC-DIR is a fixed set of iterations *N*_*fixed*_.

**Algorithm 1** CC-DIR in 3D

**Inputs:**

 **p**_*col*_, **p**_*reg*_ ∈ *R*^*i*,*j*×3^ with **p**_*col*_, **p**_*reg*_ ⊂ *ω*_*r*_ $\Phi_{R},\Phi_{C}\in R^{3\times n_{1}\times n_{2}\times n_{3}}$

**Initialize:**

 *N* ← 0

$\Phi_{R}^{0}\leftarrow 0_{3\times n_{1}\times n_{2}\times n_{3}}$

$\Phi_{C}^{0}\leftarrow 0_{3\times n_{1}\times n_{2}\times n_{3}}$ $u^{0}\leftarrow 0_{3\times n_{1}\times n_{2}\times n_{3}}$

**while** *N* ≤ *N*_*fixed*_ **do**

$\Phi_{R}^{N+1}\leftarrow \text{arg}\;{\text{min}}_{\Phi_{R}}F(\Phi_{R},I,J)+\frac{\rho }{2}{\left\|\Phi_{R}-\Phi_{C}^{N}+u^{N}\right\|}_{2}^{2}$

 $\Phi_{C}^{N+1}\leftarrow \text{arg}\;{\text{min}}_{\Phi_{C}}C(\Phi_{C},I,J)+\frac{\rho }{2}{\left\|\Phi_{R}^{N+1}-\Phi_{C}+u^{N}\right\|}_{2}^{2}$

 $u^{N+1}\leftarrow u^{N}+\Phi_{R}^{N+1}-\Phi_{C}^{N+1}$

**end while**

### Synthetic experiments

In order to show the improvements in registration quality by the CC-DIR compared to methods without collision constraining, the synthetic experiments include a Baseline model, a modified Baseline (mod. Baseline) model for improved performance and the CC-DIR. The Baseline model consists of a B-spline FFD registration model with one deformation field and a gradient descent with backtracking as optimization method. The mod. Baseline uses the same setup, however running two FFD registrations consecutively, one for each image domain. The CC-DIR computes one deformation field for each image domain as well. However, CC-DIR uses Alg. 1 to constrain collisions between the two domains. Differences in algorithms between Baseline, mod. Baseline and CC-DIR are kept to a minimum by using the same similarity measure SSD. A small parameter study has been performed for every experiment to identify the best outcome for the synthetic setup with respect to control point grid scale, learning rate, and penalty parameter.

#### Data

**Fig 1:**
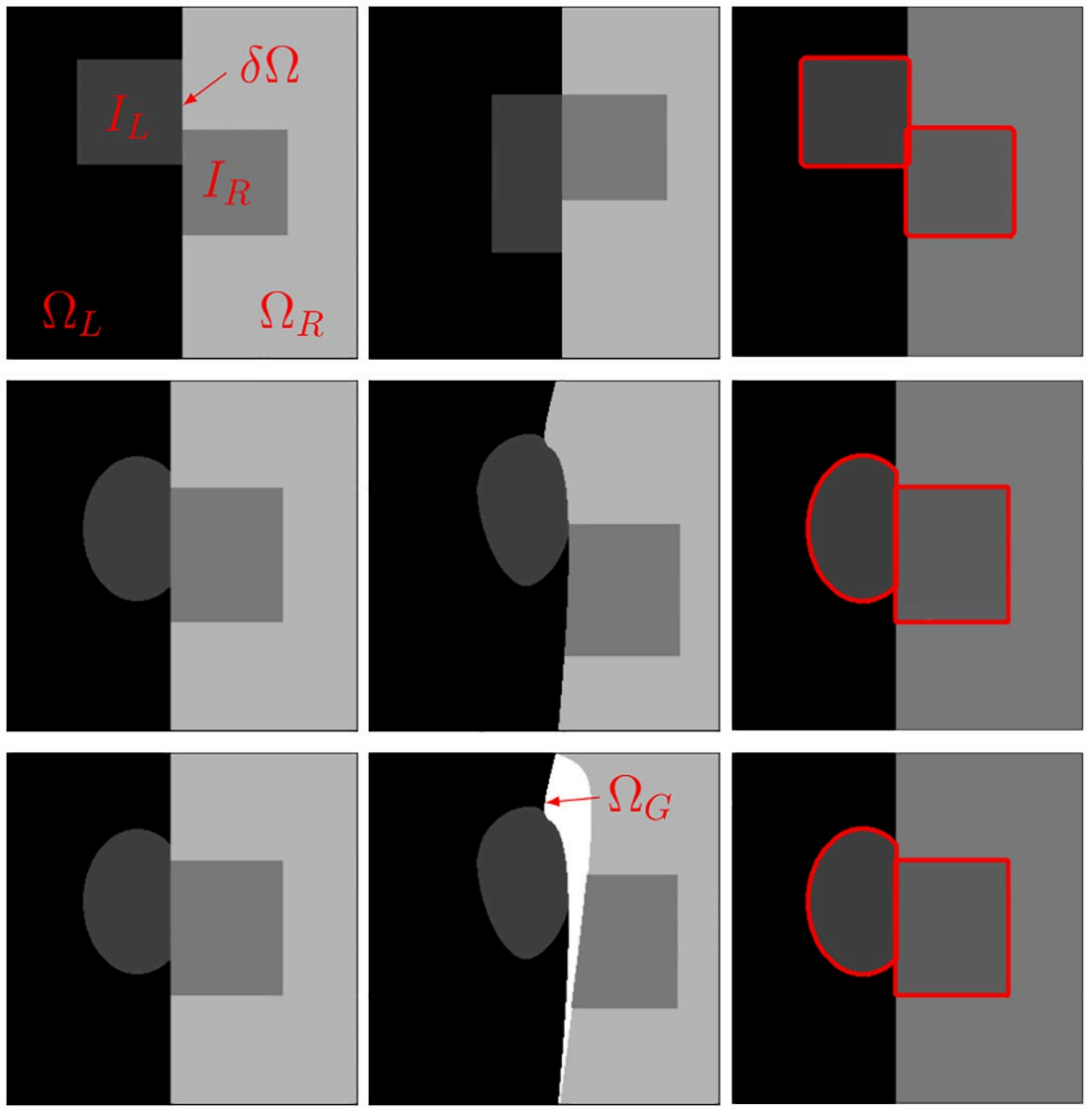
Synthetic experiments setup and results. From left to right: Source, target and pullback-registered CC-DIR images for Linear (Top row), Nonlinear (Center row) and Growth (Bottom row) case. The registered images show clear matching of the shapes *I*_*L*_ and *I*_*R*_.

Three simplified cases in 2D are computed for a better understanding and visualization of the proposed method, while combining different discontinuities in their respective setups. The image domain is divided by the boundary *δΩ* into Ω_*L*_ on the left side and Ω_*R*_ on the right, each containing the distinct structure *I*_*L*_ respectively *I*_*R*_. In the first case, *Linear*, the boundary *δΩ* moves as a whole to the right-hand side while *I*_*L*_ and *I*_*R*_ are sliding in opposite directions up, respectively down. Additionally, *I*_*L*_ deforms under conservation of its total area. The second case, *Non* − *linear*, introduces a more complex, non-linear deformation of the boundary *δΩ*. Both structures are subject to deformation and move along the boundary in contrary directions, again sliding along the boundary. The last case *Growth* combines deformation, translation and the sliding motion discontinuity from the second example with a growth Ω_*G*_ in the boundary region (Fig 1).

#### Results

Two main criteria are quantified and evaluated: registration success and collision constraint violation. The registration success is easily calculated with the Sørensen-Dice index (DICE) for matching the structures *I*_*L*_ and *I*_*R*_. For the evaluation of the collision constraint the intersection scores (IS) is introduced. The percentage of intersecting pixels with respect to the image area constitutes to IS, aiming to giving an qualitative overview of the intersection. For all three cases, the CC-DIR performs best in terms of registration as well as preventing intersection (Table 1). The DICE score indicates a high success in matching the structures, backed by a clean pullback registration visualization in Fig 1. No pixel intersects with the boundaries, so no violation of the collision constraint is detected. The mod. Baseline ranks in second, with satisfying registration results in all but the *Growth* case. Regarding intersections, this approach performs similarly well for the *Linear* case as the CC-DIR, but shows more intersections with increasing non-linearities in the boundaries. Losing registration performance quickly, the Baseline approach struggles to match the structures already in the *Linear* case. Additionally, intersections are reported for every case, hence violating the collision constraints consistently.

**Table 1:** Intersection (IS) and DICE score for synthetic experiments. The purposed CC-DIR based algorithm shows no overlap in the three test cases additionally with a superior DICE score.

*Experiment*	*Mode*	*IS [%]*	*DICE*
Linear	Baseline	0.15	0.954
	mod. Baseline	**0.0**	0.976
	CC-DIR	**0.0**	**1.0**
Non-linear	Baseline	0.2	0.98
	mod. Baseline	0.14	0.984
	CC-DIR	**0.0**	**0.998**
Growth	Baseline	3.48	0.843
	mod. Baseline	3.44	0.876
	CC-DIR	**0.0**	**0.999**

### Medical experiments

The CC-DIR for evaluation on a medical data set is run as described in Alg. 1. Two deformation fields are computed consecutively and independently. The two independent domains of *I* ∈ *R*^3^ are the lung domain Ω_*Lung*_ ⊂ *I* defined by a segmentation and the rib cage Ω_*Rib*_ = *I*\*Omega*_*Lung*_. The multiresolution procedure is derived from the findings in the EMPIRE challenge [41]. For the best possible results, a possible initial affine registration, followed by a multiresolution CC-DIR registration is run. The multiresolution setup consists of multiple successive CC-DIR registrations, with a coarse-to-fine control point grid adaption. The successively run CC-DIR compute updated starting coordinates for *p*_*col*_, *p*_*reg*_, handing these down to the next finer resolution CC-DIR. Image similarity is measured by SSD. Mirroring the synthetic experiments, the registration success and collision constraint violation are evaluated. The results are compared to a set of algorithms also tackling the sliding motion along the lung-ribcage interface [14].

#### Data

For the medical test setup, the DIR-LAB 4DCT data set from [42] is used with previously segmented lung masks from the Continuous Registration Challenge [43]. The registration is computed between the extreme inhale-exhale image pairs in order to ensure high deformations and sliding motions. To exclude regions with little image inforamtions, the images are cropped to exclude inmaging artifacts at the boundary regions. All CC-DIRs are run for 100 iterations.

#### Parameters

Even though consisting of only 10 lung scans, the DIR-Lab data set contains a variety of motion patterns, requireing individually tuned parameters. Hence, initial affine registrations were run for cases 5-10 as a gradient descent with backtracking for 100 iterations and SSD for similarity measure. The multiresolution scales in mm for the (x,y,z) axis for cases 1, 6, 7, 9, and 10 were (19.4,19.4,50), (9.7,9.7,25), and (4.85,4.85,12.5). Cases 2 had scales (23.2,23.2,50), (11.6,11.6,25), and (5.8,5.8,12.5). Case 3 had scales (23,23,50), (11.5,11.5,25), and (5.75,5.75,12.5). Case 4 used (22.6,22.6,50), (11.3,11.3,25), (5.65,5.65,12.5) as scales and case 5 (22,22,50), (11,11,25), (5.5,5.5,12.5). And finally case 8 with (38.8,38.8,100), (19.4,19.4,50), (9.7,9.7,25), and (4.85,4.85,12.5).

#### Target registration error

For the registration accuracy, anatomical landmarks are used. The data-set provides 300 annotated landmarks in both extreme phases. Consequently the summed position error after registration, called target registration error (TRE) over all landmarks indicates the registration outcome (Table 2). Introduced in the original data set publication [1, 20, 42], a snap-to-voxel evaluation for the TRE is used. The proposed CC-DIR algorithm’s overall TRE accuracy ranks at fourth place, while matching the best benchmark in cases 1 and 2.

**Table 2:** Comparison of TREs for the DIR-LAB data set in mm. The mean TRE of the purposed CC-DIR method ranks at fourth place out of seven with an offset of 0.15 mm to the best performing algorithm.

Case	Wu (2008)	Delmon (2013)	Berendsen (2014)	Hua (2017)	Eiben (2018)	Gong (2020)	Proposed CC-DIR
1	1.1 ±0.5	1.2 ±0.6	1.0 ±0.52	1.0 ±0.51	N/A ±N/A	0.97 ±N/A	**0.92±0.97**
2	1.0 ±0.5	1.1 ±0.6	1.02 ±0.57	0.99 ±0.59	N/A ±N/A	1.02 ±N/A	**0.9±1.14**
3	1.3 ±0.7	1.6 ±0.9	1.14 ±0.89	**1.12±0.64**	N/A ±N/A	1.18 ±N/A	1.23 ±1.43
4	1.5 ±1.0	1.6 ±1.1	1.46 ±0.96	1.44 ±1.03	N/A ±N/A	**1.38±N/A**	1.49 ±1.41
5	1.9 ±1.5	2.0 ±1.6	1.61 ±1.48	**1.37±1.35**	N/A ±N/A	1.45 ±N/A	1.9 ±2.48
6	1.6 ±0.9	1.7 ±1.0	1.42 ±0.89	1.26 ±1.04	N/A ±N/A	**1.12±N/A**	1.46 ±1.41
7	1.7 ±1.1	1.9 ±1.2	1.49 ±1.06	**1.12±0.67**	N/A ±N/A	1.24 ±N/A	1.42 ±1.43
8	1.6 ±1.4	2.2 ±2.3	1.62 ±1.71	**1.18±1.22**	N/A ±N/A	1.71 ±N/A	1.26 ±1.17
9	1.4 ±0.8	1.6 ±0.9	1.3 ±0.76	**1.14±0.64**	N/A ±N/A	1.24 ±N/A	1.2 ±1.06
10	1.6 ±1.2	1.7 ±1.2	1.5 ±1.31	**1.08±0.82**	N/A ±N/A	1.24 ±N/A	1.45 ±1.72
Mean	1.47 ±0.96	1.66 ±1.14	1.36 ±0.99	**1.17±0.85**	1.21 ±N/A	1.25 ±N/A	1.32 ±1.42

**Fig 2:**
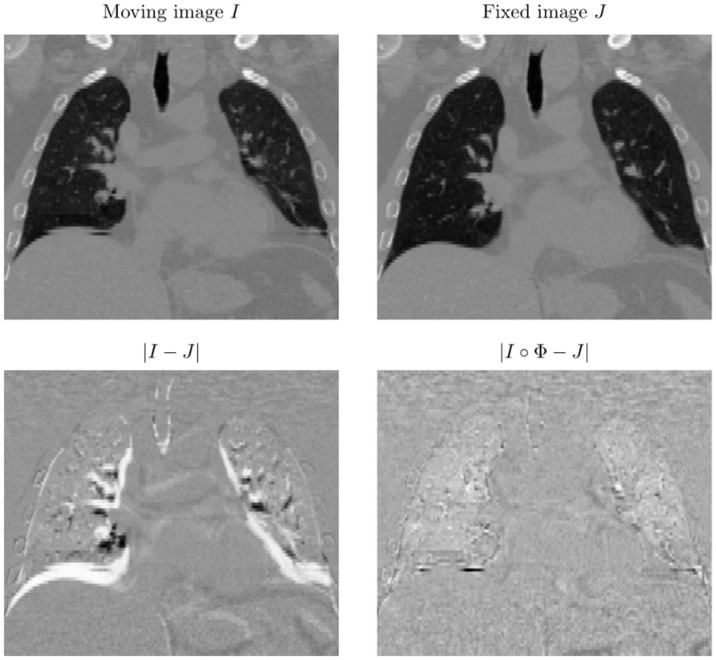
Overview of the reference, source and registered image. |*I* − *J*| shows the intensity differences between moving and fixed image without registration, |*I*∘Φ − *J*| after collision-based registration. A clear reduction of intensity differences and matching of anatomical structures can be observed.

**Fig 3:**
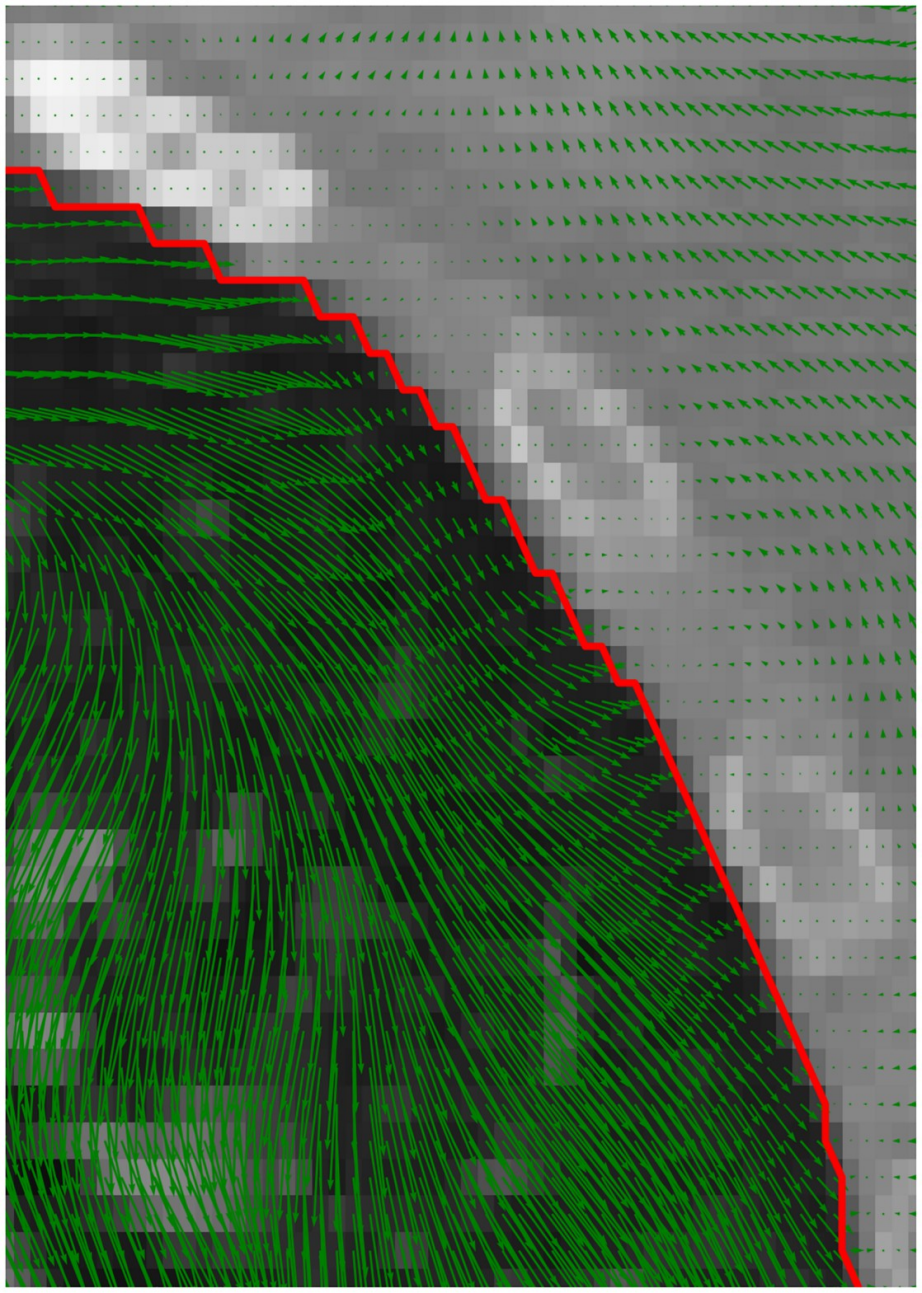
Close-up of the displacement field. Displacements around the interface (green arrows) show observable sliding motion along the boundary (red).

A coronal slice of a 3D CT lung scan in Fig 2 shows an example of the registration process. The difference image between the moving and target configuration before and after registration depict a reduction of structural differences. The deformation grid overlaid over the registered image in Fig 3 is the result of the CC-DIR. This close-up shows a clear solution to the sliding interfaces along the pleura as well as a translation in the diaphragm caused by the inhalation.

#### Congruent interfaces

For the evaluation of congruent, and thus physiologically plausible, rib-cage-lung interfaces, the metric introduced in [24] is used. After transforming a 3D surface mesh of the rib-cage-lung boundary *δΩ* by both displacement fields Φ_*Lung*_ and Φ_*Rib*_, the subsequent gap and overlap between the two domains is calculated by voxelizing the respective meshes. The results in *cm*^3^ are recorded in Table 3.

**Table 3:** Comparison of gap/overlap for the DIR-LAB data set by case. The purposed CC-DIR algorithm shows the least overlap compared to the six other methods. It rates second in terms of interface gap with a mean offset of 38.1 *cm*^3^ to the first place. All measurements are in *cm*^3^.

Case	Wu (2008)	Delmon (2013)	Berendsen (2014)	Hua (2017)	Eiben (2018)	Gong (2020)	Proposed CC-DIR
1	38/26	39/15	23/18	39/**2**	N/A/N/A	67/69	**22**/2
2	78/46	67/60	74/34	74/31	N/A/N/A	76/94	**53**/**6**
3	99/28	83/33	57/30	71/24	N/A/N/A	59/70	**53**/**7**
4	75/34	66/44	66/28	92/13	N/A/N/A	71/79	**41**/**7**
5	**11**/38	78/52	61/32	54/**6**	N/A/N/A	97/109	37/11
6	**10**/86	119/77	130/50	155/**11**	N/A/N/A	57/69	90/23
7	**10**/79	108/77	119/45	138/19	N/A/N/A	73/88	131/**14**
8	96/91	92/93	85/53	150/40	N/A/N/A	**80**/98	92/**39**
9	61/34	**54**/44	70/51	58/14	N/A/N/A	63/72	78/**8**
10	120/63	94/56	80/43	109/28	N/A/N/A	90/110	**48**/**19**
Mean	88.2/52.5	80.0/55.1	76.5/37.4	94.0/18.8	**26.4**/34.5	73.3/85.8	64.5/**13.6**

Overall, and in half of the cases specifically, the novel CC-DIR registration shows the least amount of overlap. The average resulting gap between the two domains ranks second after the interface matching algorithm [20].

#### Shear

In order to estimate the shear along the domain interfaces, the maximum shear stretch introduced in [44] is calculated. Using the deformation gradient tensor $F=\frac{dx}{dX}$ which maps the transformation from the undeformed configuration *x* to the deformed state *X*, the maximum shear stretch constitutes to

$$\begin{array}{c} \lambda_{max}=\frac{\lambda_{1}-\lambda_{3}}{2} \end{array}$$ (11)

with

$$\begin{array}{c} \lambda_{i}=\sqrt{eigenvalues\;of\;F^{T}F}. \end{array}$$ (12)

**Fig 4:**
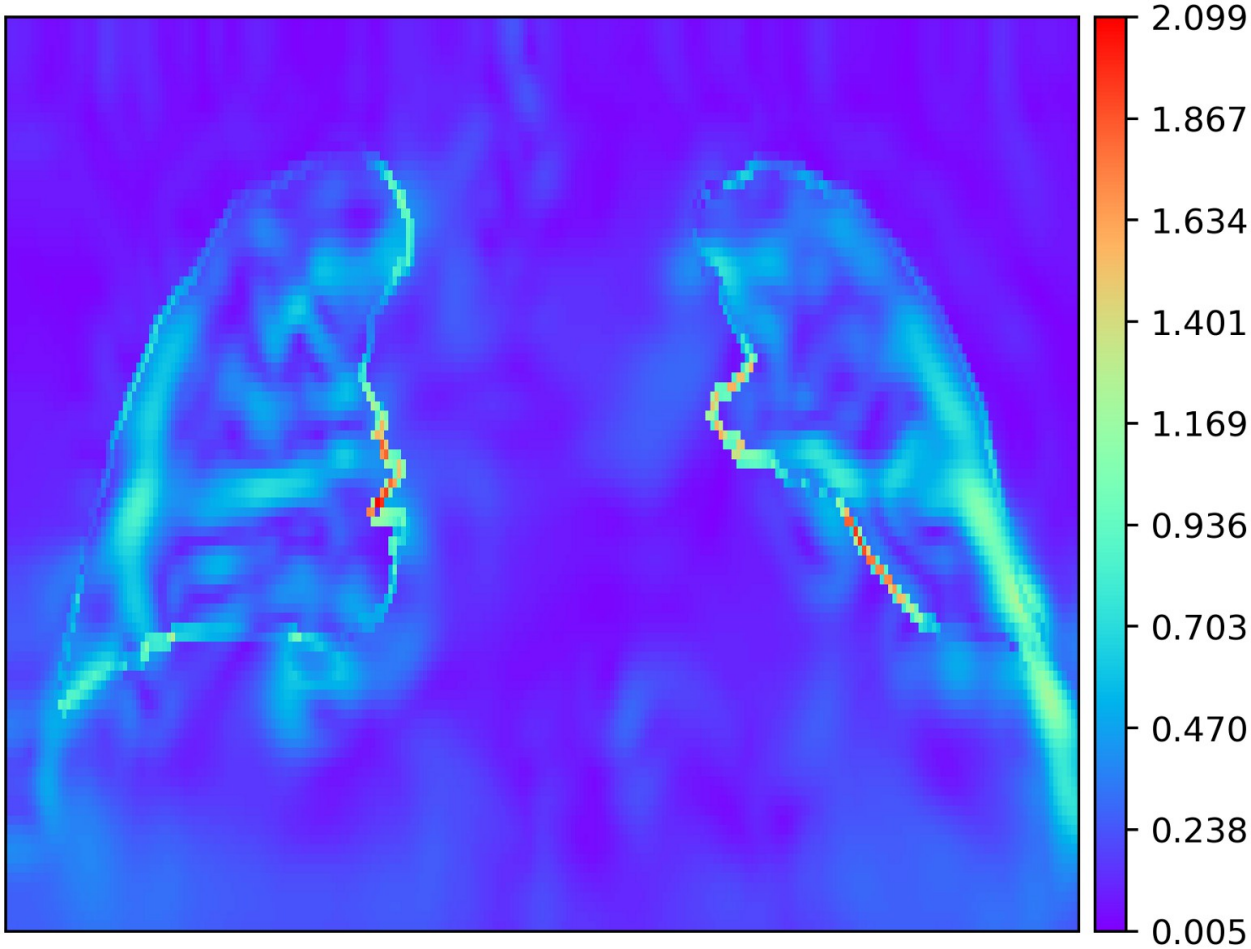
Shear stretch evaluation. High differences in shear stretches along lung-rib-cage interface with respect to the rest of the image are depicted.

The exemplary colour-coded representation Fig 4 clearly shows an increase in shear stretches along the interface, whereas the displacement field in Fig 3 demonstrates sliding motion. Overall the mean shear stretches amount to 1.3 ± 1.8 between the lung and rib-cage, respectively 0.2 ± 0.2 in the rest of the image. The computation time averages at 822s ± 519s on a Xeon CPU @ 3.60GHz x12 with an GPU implementation on a GeForce RTX 3090.

## Discussion

Coupling deformable image registration with collision detection offers up new possibilities for supplying registration algorithms with simple physics. In order to incorporate these physics into the common registration problem formulation, an energy term (Eq 7) is needed. This setup simulates the deformation of the distinct image domains, such as organs, by calculating separate deformation fields. Discontinuities at the domain boundaries, such as sliding motion don’t need to be modelled explicitly but result automatically by the collision forces minimizing the non-intersection constraint. Setting up the collision detection requires a domain representation, either as segmentation masks, surface or volumetric meshes. Acquiring these representations can be a difficult task, especially for regions with no discernible or low-contrast domain boundaries. A problem encountered in the experiments is the lack of image information at the domain boundaries. If registration is strictly applied to the respective domain masks, the resulting gap might increase as image information outside of the sub-domain is missing. Dilating the masks to increase the tracking image forces provided an acceptable alternative. This way extensive domain overlap post-registration is prevented by the collision constraints, whereas possible gaps between the domains are closed by the image forces. Compared to single deformation field methods, CC-DIR allows to easily use different parametrizations and parameters for different sub-domains. Since no costly on the run identification of the domain boundary, as in filter based methods, has to be performed, the possibility of surface errors in low contrast regions is avoided. Not relying on normal and tangential decomposition also permits solving sliding motions along complex and non-smooth surfaces. Unless other mentioned methods registering subdomains independently using intensity based constraints, collision detection is geometric and thus modality independent, allowing the collision detection and correction to be used in different settings. Since collision detection is a fundamental area in computer graphics, it can fall back on a considerable body of research and a variety of algorithms, permitting an exchange of the collision detection method, as long as a respective energy term is formulated. An advantage of using collision detection over interface matching methods is the circumvention of redundant constraints, letting the registration energy term drive the deformation. Additionally, collision detection can be applied to scenarios where subdomains are moving freely around the image domain, with no restriction to constant contact between the subdomains, such as in computer vision or robotics. The implementation of the collision constraint in the ADMM has two advantages: First, the modularity of this optimization scheme permits not only the exchange of the collision method but also of the registration method specifications such as similarity metric or deformation model at will. Additional and existing regularization models can be freely and effectively added to the registration energy term, subsequently offering sliding motion representation at no further cost. Second, since the penalty function 6 is not bound to be differentiable, the ADMM still converges [39] and avoids performance issues of gradient-based methods [1]. However, as with many optimization strategies, convexity is of critical importance. First, the convergence of the ADMM is formally mathematically proven for convex functions only. Second, the ADMM solves the minimization for the dual problem instead of the initial primal problem. If strong duality holds, usually for convex functions [45], the dual solution is the primal solution [39]. The obtained solutions from nonlinear and non-convex problems, such as registration, might offer a lower bound and if converged, the ADMM still offers local solutions [39]. Furthermore, with additional regularization, the convexity of the objective functions might be increased and thus reduce the duality gap.

Looking at the results from the experiments in detail, more practical observations can be made. The synthetic experiment setups were chosen to analytically examine the possible improvements of the CC-DIR with collision detection compared to registration algorithms without collision detection. The results from the synthetic experiments show with stark contrast the benefits of the collision detection modeled via the CC-DIR compared to the Baseline approaches. The CC-DIR outperforms the Baseline approaches not only in terms of intersection constraints but also with respect to registration. Additionally to enforcing the non-intersection constraint, the collision detection acts as a powerful regularization term. Since the registration loss functions are likely to be non-convex, this regularization might increase convexity at least locally if not globally. Regarding the intersection constraints, the CC-DIR delivers deformation fields with no intersection at all, while the Baseline approaches have difficulties adhering to these boundaries.

Looking at the registration success for the medical data set measured by the TREs, this algorithm ranks in fourth out of seven, with an average distance on 0.15 mm to the best ranking algorithm. However, with the finest voxel resolution of 0.97mm in the DIR-Lab data set, these differences can be regarded as minimal and may be caused by the image discretization. Furthermore, due to its advantageous framework build, replacing the registration methods is not only simple, but any improvement to the registration methods will also likely increase the TRE accuracy. With the least overlap and the second lowest gap between the domains compared to other algorithms, the CC-DIR produces separate yet congruent deformation fields that minimize physiologically not feasible transformations. Even though the CC-DIR doesn’t prevent overlapping completely, two adaptions may offer an improvement: individually tuned hyper-parameters and an alternative constraint formulation. The implementation as quadratic penalty functions has the possible disadvantage of producing inexact solutions as the iterates may be drawn to points that violate the equality constraints but satisfy optimality conditions [37]. Exchanging the quadratic penalty function with an exact penalty function might yield the desired solution, however the resulting non-smoothness should be taken into account.

## Conclusion

The CC-DIR successfully introduces a collision constrained method into the field of image registration. With its simple framework build, it allows to combine state-of-the-art registration methods with collision detection for discontinuity preservation. No major drawback in terms of registration quality have been observed, thus providing an effective method to account for sliding motion while simultaneously ensuring congruent interfaces. Inspired by the results in this paper, a future development of this framework is the exchange of the registration method with diffeomorphic counterparts. Looking at image registrations of the liver or prostate, which frequently utilize the Finite-Element-Method to simulate deformations [46], an image driven pathway to simulations may open up new possible developments. The advantageous movement parametrization based on basis functions can be used to easily derive FE models. A reformulation with tetrahedral interpolation and movement parametrization coupled with collision detection between tetrahedral meshes and deformable signed distance fields can even boost this process to fuse image registration with bio-mechanical analysis.
